# *‘Deep down in their heart, they wish they could be given some incentives’*: a qualitative study on the changing roles and relations of care among home-based caregivers in Zambia

**DOI:** 10.1186/s12913-015-0685-7

**Published:** 2015-01-28

**Authors:** Fabian Cataldo, Karina Kielmann, Tara Kielmann, Gitau Mburu, Maurice Musheke

**Affiliations:** Dignitas International, Research Department, Zomba, Malawi; Institute for International Health and Development, Queen Margaret University, Edinburgh, Scotland; Independent Consultant, New York, NY USA; International HIV/AIDS Alliance, Brighton, UK; Division of Health Research, Lancaster University, Lancaster, UK; Zambia AIDS Related Tuberculosis Project, University of Zambia, Lusaka, Zambia

**Keywords:** Home-based care, Community health workers, HIV, Motivation, Zambia

## Abstract

**Background:**

Across Sub-Saharan Africa, the roll-out of antiretroviral treatment (ART) has contributed to shifting HIV care towards the management of a chronic health condition. While the balance of professional and lay tasks in HIV caregiving has been significantly altered due to changing skills requirements and task-shifting initiatives, little attention has been given to the effects of these changes on health workers’ motivation and existing care relations.

**Methods:**

This paper draws on a cross-sectional, qualitative study that explored changes in home-based care (HBC) in the light of widespread ART rollout in the Lusaka and Kabwe districts of Zambia. Methods included observation of HBC daily activities, key informant interviews with programme staff from three local HBC organisations (n = 17) and ART clinic staff (n = 8), as well as in-depth interviews with home-based caregivers (n = 48) and HBC clients (n = 31).

**Results:**

Since the roll-out of ART, home-based caregivers spend less time on hands-on physical care and support in the household, and are increasingly involved in specialised tasks supporting their clients’ access and adherence to ART. Despite their pride in gaining technical care skills, caregivers lament their lack of formal recognition through training, remuneration or mobility within the health system. Care relations within homes have also been altered as caregivers’ newly acquired functions of monitoring their clients while on ART are met with some ambivalence. Caregivers are under pressure to meet clients and their families’ demands, although they are no longer able to provide material support formerly associated with donor funding for HBC.

**Conclusions:**

As their responsibilities and working environments are rapidly evolving, caregivers’ motivations are changing. It is essential to identify and address the growing tensions between an idealized rhetoric of altruistic volunteerism in home-based care, and the realities of lay worker deployment in HIV care interventions that not only shift tasks, but transform social and professional relations in ways that may profoundly influence caregivers’ motivation and quality of care.

## Background

Recent years have seen renewed interest in the potential of community health workers (CHWs) to expand access to essential health care services, particularly in light of critical shortages in the health workforce [1]. CHWs have featured prominently within discussions of task-shifting initiatives to address the human resources for health crisis [2], most notably in the context of ‘roll-out’ and ‘scale-up’ of HIV treatment and care programmes [3].

With widespread availability of antiretroviral therapy (ART) across Sub-Saharan Africa since the early 2000s, HIV care has shifted from clinical and palliative management of a terminal condition to largely home-based and life-long support of people living with a chronic condition [4]. Community and lay health care workers are increasingly involved in delivering HIV care, and have a role to play in improving patient outcomes and reducing the work burden of health workers [5].

In Zambia, CHWs have long been involved in providing support to the health system: Zambia was one of the first countries in sub-Saharan Africa to implement home-based care (HBC) for people living with HIV and AIDS (PLHIV) in the early 1990s. While a number of faith and non-governmental organizations (NGOs) in Zambia provided counselling, nursing and palliative care when treatment was unavailable, the tasks undertaken by home-based caregivers have evolved considerably along the demands of testing and treating PLHIV, and sustaining individuals on long-term treatment. Despite their long-standing role in HIV care, CHW programmes in Zambia have faced challenges related to low motivation, inadequate supervision, insufficient compensation, and the lack of recognition by formal health providers [6,7]. A comprehensive community health policy and strategy to guide the operations of this workforce is yet to be put into effect in Zambia; regulation or monitoring of home-based care services has generally been left to individual institutions or organisations engaging the CHWs [6]. Recent efforts at establishing Community Health Assistant programmes in Zambia have faced criticism for ‘destroying’ the culture of volunteerism and fuelling conflict with existing lay CHWs [6].

The evidence from Zambia adds to the growing body of public health literature examining the broader feasibility and functional challenges of integrating CHWs into national health systems [7,8] as well as a more narrowly conceived objective of task-shifting in the context of ART roll-out [9-13]. However, little attention has been given to the impact of such initiatives on organisational culture and on the intrinsically hierarchical social and professional divisions that characterise real-life health systems. A number of social scientists have, on the other hand, documented the effects of global health reforms and initiatives on different types of CHWs, highlighting their simultaneous ‘embeddedness’ yet liminality within evolving health systems and their commonly shared experiences of conflicting accountabilities vis-à-vis task-oriented bureaucracy and patient demands [14-17]. Social scientists have highlighted the need to situate CHWs’ motivation, recruitment and retention within complex socio-political contexts [18] in which CHWs occupy a structural position of marginality between formal and informal labour [19]. These studies offer compelling insights into individual trajectories of frontline health workers in resource-constrained settings. Yet they also reflect the tensions between emotional work and unpaid labour that caregiving in these contexts represents. These tensions are essentially about the value placed on different relations of care within the health system. While care is inherently relational for social scientists, and consequently, ‘good care’ often linked to ‘good social relations’ , this observation is far less evident for health systems researchers.

We argue that an emphasis on social relations of care has been missing from the literature reviewing the effectiveness of community-based strategies for the scale up of priority public health interventions including ART. Strategies increasingly rely on what is seen to be a more rational or coordinated deployment of human resources of health (HRH) through partnerships, integration, substitution, or task-shifting initiatives. These arrangements inevitably impact on health workers’ skills and status, their working culture, and their relationships with patients – and the ensuing changes in relationships may both encourage and hinder the motivation to provide good care. Drawing on data from a qualitative study conducted with home-based caregivers in Zambia in 2009, our paper uses the devolution of ART access in Sub-Saharan Africa to explore how relations of care evolve alongside the scale-up of health interventions, which may also inform other public health interventions involving lay workers.

## Methods

This paper draws on data collected in the context of a qualitative study conducted in Zambia from November 2008 to July 2009. The research examined changes in HBC for PLHIV in the context of increasing availability of ART in Zambia from the mid-2000s. The objectives of the study were two-fold. First, we aimed to document how the roll-out of ART affected the scope and the nature of caregivers’ working practices and their status within the formal health system. Second, we examined how the changes in roles and responsibilities within the mandate of ART delivery affected home-based caregivers’ relationships with PLHIV and their families.

### Study design

The research was cross-sectional and observational, drawing on qualitative methods including field observations and in-depth as well as key informant interviews.

### Study setting

The study was conducted over a period of one year within three HIV-related community based organisations (CBOs) providing HBC services: the *Family Health Trust* in Lusaka; *Kara Counselling* and *Dackana Community Home Based Care and OVC Support* in Kabwe. One member of the research team conducted an initial scoping activity to profile eight CBOs providing HBC for PLHIV in Lusaka, Monze, Kapiri Mposhi and Kabwe districts, gathering information on size, years of experience, structure, number of caregivers, and HBC activities. We then approached the managers of the three CBOs that had been established for at least four years prior to start of the fieldwork, and had extensive experience in the provision of HBC services to PLHIV, including facilitating access to ART in the Lusaka and Kabwe areas. The selection of the three organisations also took account of location and catchment areas (rural and peri-urban) covered by the CBOs.

### Participant selection

Two research assistants were based in Lusaka and Kabwe respectively during the length of data collection. Over the course of data collection, they familiarised themselves with the day-to-day activities of the three CBOs, for example, by attending staff meetings and meeting informally with home-based caregivers. This allowed for opportunistic and snowball sampling techniques to identify research participants in each site. The study population comprised of CBO programme staff (n = 17), home-based caregivers (n = 48), and PLHIV (n = 31) affiliated with the three CBOs, as well as ART staff (n = 8) from public health facilities in the two study sites. PLHIV (HBC clients) were identified through their links with HBC activities, i.e. having received HBC services and continuing to receive some form of support from a home-based caregiver. Finally, ART staff (nurses and clinical assistants) was identified within public health facilities linked to HBC programmes in Lusaka and Kabwe.

### Data collection methods

Qualitative data was collected over a period of eight months (2008–2009). At the outset of the study, the investigators and research assistants conducted field visits and key informant interviews (KII) with HBC programme staff and ART clinic staff. The KII served to understand more about the local context, history, and changes within HBC in the two study sites, as well as perceptions of the role of home-based caregivers in HIV care. Clinic staff members were asked specifically about ART-related services provided by the clinic, the interface between the ART clinic and other departments of the health facility and HBC services, as well as the ART treatment pathway, including resort to CHWs and HBC.

In-depth interviews were held with home-based caregivers and PLHIV to capture the experiences of giving and receiving care in the context of ART. In-depth interviews with caregivers explored their motivations, training received, the perceived nature of HBC and the type of HBC support provided, positive and negative impact of ART services on the delivery of HBC, interface with formal health care providers, and the challenges of delivering HBC. Interviews with PLHIV aimed to elicit information on individual experiences of seeking HIV testing, accessing ART, and being on treatment; the different forms of support received whilst on treatment including the role of HBC providers in treatment support; and the perceived changes in HBC with the onset of ART service provision.

Interviews were conducted in English, Bemba and Nyanja, and took place in a private area chosen by the respondent. They lasted between 45 minutes and one hour. Respondents were provided with information about the study and invited to participate. If willing, informed consent was taken before the interviews and observations. The interviews were audio-recorded with the permission of the interviewee.

The research assistants also conducted observations on a daily basis and recorded field notes. Observation notes involved reflections on the research assistants’ interactions with home-based caregivers, and participation in activities of the HBC organisations, for example, accompanying caregivers during their home visits and attending weekly meetings at the three CBOs. In Kabwe, the research assistant attended weekly planning meetings, monthly trainings sessions/workshops as well as local events.

### Data analysis

Interviews were transcribed and translated verbatim to English. Field observations were typed up. Data were anonymised and pseudonyms were used to protect the identity of research participants. An analysis framework was established through an integrated approach [20] that employed both deductive as well as inductive reasoning in order to identify themes that resonated with the original study questions as well as more analytical themes that emerged through repeated review, discussion and interpretation of the data among the study team. The following broad descriptive categories were used to organise the data: profile of patients on ART; description of caregivers; scope/level of activity of HBC before and after ART; status and relationships of caregivers with clients and public health sector; and impact of HBC on the health and lives of people living with HIV. More analytical sub-themes were identified in relation to inter-personal processes and dynamics of care, relating, for example, to caregivers’ personal intentions, motivations, and the content of social interactions with the care recipients. The transcripts were entered into NVivo8 and the coding framework previously developed was applied to organise the data. The preliminary study results were presented and discussed with home-based caregivers and CBOs staff during a meeting organised in Kabwe at the end of the data analysis. Feedback from research participants was included in the study report and recommendations provided to local stakeholders.

### Ethical approval

The study was approved by the Ethics Committees of both the University of Zambia and the London School of Hygiene and Tropical Medicine. Clearance to conduct interviews with ART staff in the public health facilities in the study sites was also obtained from the Zambian Ministry of Health, at national and district levels.

## Results

The study results are presented in three main sections. In the first two sections, we explore how the expansion of the ART programme in Zambia affected home-based caregivers’ working practices and their professional status within the formal health system. In the third section, we describe how the changes in roles and responsibilities of HBC providers affected caregivers’ relationships with PLHIV and their families.

### Shifting roles in HIV care

Against a backdrop of chronic poverty, households affected by HIV suffer multiple crises. The tragedy of losing family members over generations, higher numbers of dependents, and recurrent illness translate into arduous challenges of ‘getting by’ on meager incomes and weak social ties. Although the majority of the interviewed PLHIV (n = 20) were between 30 and 50 years of age, only a few (n = 4) were in formal employment. Even with the availability of ART, many PLHIV referred to the reality of food shortages and the mental, social and financial stresses associated with having a life-long condition as overwhelming challenges.

At the same time, HBC clients and caregivers witnessed a transformation of their personal and professional circumstances respectively since the advent of ART availability in the mid-2000s. Many of the 31 PLHIV interviewed in the study talked about how accessing treatment was linked to regaining health and being able to resume functional activities:

When I started ART, I quickly felt well, I gained some strength and stopped feeling ill […] I can do all these house chores and walk without feeling any pain, I am now fine (HBC client with Kara Counselling, Kabwe, 1–9).

With their clients’ increasing self-reliance, caregivers noticed a marked shift in their own roles, from hands-on physical patient care and supportive activities within the household such as cleaning and cooking to medicalised tasks geared towards enabling clients to understand, access, and adhere to ART. Most of the 48 caregivers interviewed commented on the contrast between pre- and post ART chores; the following quote is typical of this cohort:

Before they [HBC clients] started ART, we had a tough time, because every home we went to we would find that one is bedridden and your work was so hard… you will need to lift him […] but now since everyone is on their feet, it means that our job is a little bit lighter (Home-based caregiver from Family Health Trust, Lusaka 1–5).

The need for more specialised care put new demands on the competencies of home-based caregivers. Caregivers mentioned how CBOs and NGOs strengthened their skills through specialised training. For example, most caregivers received instructions on managing clients on ART as ‘treatment supporters’ and ‘patient monitors’ – these roles involved regularly following up on clients, counselling them to adhere to treatment, checking that they took the drugs as prescribed, referring new patients to the clinics, and tracing those who did not attend appointments. For a far smaller number of caregivers, an expanded set of tasks was more directly related to the technical support of patients on ART: picking up and delivering drugs for clients when necessary and identifying and diagnosing health conditions, especially those related to ART and its side effects.

Gaining more specialised knowledge was a motivational factor for many caregivers interviewed. Some accounts of training allude to the manner in which knowledge acquired enabled caregivers to set themselves apart from ‘traditional’ help-seeking pathways:

Kara Counselling has taught us a lot of things. We never knew anything about STIs called Syphilis and Gonorrhoea. […] We used to search for help from traditional healers. […] We are [now] able to recognise symptoms and interact with an [HIV-infected] individual with an intention of counselling or persuading them not to waste their time visiting traditional healers”. (Home-based caregiver with Kara Counselling, Kabwe 1–6).

At the same time, the acquisition of new skills by caregivers was accompanied by growing expectations of monetary and material rewards as home-based caregivers came to see themselves as fulfilling the work of paid formal health care workers. The lack of financial and material compensation for HBC work was seen by the programme representative for one of the CBOs (Family Health Trust, Lusaka) to have resulted in a dwindling sense of commitment among caregivers:

In most cases, when they [home-based caregivers] hear that there is some foodstuff and something good, you find that 35 or 40 will come at that particular time. When they see that there is nothing they all disappear. If they are asked why they are not available, they normally say that they have started doing other activities or they are busy cultivating, but initially those who are committed are less than 25 (HBC programme representative, Family Health Trust, Lusaka 1–10).

While HBC caregiving activities have become more aligned with the demands of ART programmes, changes in the availability of funding for HBC activities have impacted the level of material and financial support received by home-based caregivers. There is a tension between the move towards formalisation of their roles as ‘treatment supporters’ on the one hand, and diminishing visibility in terms of donor interests and lack of compensation on the other hand.

### Transforming care relations in the workplace

The uptake of activities by home-based caregivers - such as patient referrals, filling in registries, and supporting those on ART - was welcomed by the formal health staff members, who acknowledged that home-based caregivers contributed to alleviating their own burden of work. The observation a nurse-in-charge at one of Kabwe’s ART centres was widely shared amongst formal health care staff:

Home-based caregivers are able to monitor patients within their catchment areas. They are able to attend to the complaints of these clients and also to bring these complaints to the health centres so that we can attend to them. […] Their presence, we really value it, it is like relieving the burden that us health care workers are supposed to perform […] Seeing that there is a shortage of staff, […] they are doing a commendable job for us (ART clinic nurse, Kabwe, KII-1).

Staff from the ART centres saw caregivers as having a privileged ‘window on the community’. The ART clinic nurse in Kabwe describes a form of partnership in adherence support and patient monitoring:

We have to work hand-in-hand so that these patients continue their treatment. They [home-based caregivers] alert us and make us know what is taking place out there in the community: *Are patients adhering to the drugs? How many have stopped the drugs? How many have died?* These data are very important for us. (ART clinic nurse, Kabwe, KII-3).

Home-based caregivers themselves noted a change in their relationship with formal health care staff. The majority of home-based caregivers described their roles as gaining in importance as they had become closer to those of paid formal health staff:

Our working relationship with nurses in government health institutions has grown and is enhanced because we do almost similar work […] we make work lighter for health workers (Home-based caregiver with Dackana, Kabwe 1–1).

For a few caregivers, involvement in decision-making and interactions with health care staff and with clients conveyed a sense of professionalised identity:

There are moments when I receive a phone call from my client’s relatives that the sickening condition has worsened. […] I will be very busy assessing whether my client’s condition is suitable for home care or needs to be admitted at the hospice. If the condition is a hospice case, I will have to organise transport so that we can bring the client to the hospital (Home-based caregiver with Kara Counselling, Kabwe 1–3).

Increased responsibility and accountability to the formal health system had, however, did not necessarily translate into shared and recognised professional status, as expressed by most caregivers:

We do not have IDs for health care staff to identify us as caregivers […] we are not known by the health workers. […] If we had uniforms, that would help (Caregiver with Family Health Trust, Lusaka 1–14).

Home-based caregivers continued to be seen as the lowest ranking cadre of health care workers, motivated primarily by altruistic principles. The programme manager of one of the CBOs expressed a more common discourse around HBC:

Home-based care itself is based on volunteerism. So a typical HBC provider is a person who volunteers his or her time to render some services to the community […] a passionate person, able to take very good care of others (Programme manager with Dackana, Kabwe 1–2).

The organisational rhetoric of volunteerism contrasted strongly with widespread dissatisfaction among home-based caregivers with the lack of compensation received as compared to formal health workers. One caregiver was particularly vocal in reflecting these tensions:

Caregivers are often scared of talking about incentives because they fear that when they raise the issue, someone will just say: ‘but you are just volunteers, what incentives do you want?’ So some caregivers pretend that all is well, but deep down in their heart, they wish they could be given some incentives. (Home-based caregiver with Family Health Trust, Lusaka, 1–14).

As they acquired specialist knowledge in assisting clients on ART, thresholds between lay and formal care had become increasingly blurred. Increased responsibility and accountability to the formal health system has, however, not necessarily translated into professional status, as many caregivers argued that their efforts are under-valued; they lacked formal affiliation, training and remuneration.

### Evolving expectations between home-based caregivers and clients

Most home-based caregivers worked with a handful of clients, sometimes only one, and had developed long-term relationships with them and the rest of their households. They were part of their clients’ social network, often described as ‘family members’ by PLHIV and their families, and represented, for some, a trusted source of support:

If a client wants to confide in me something he doesn’t want the family members to know, we talk about it and it remains between the two of us (Home-based caregiver with Family Health Trust, Lusaka 1–4).

When I am hungry and I don’t have food, I normally go to her place [home-based caregiver’s] to ask for food and she gives some to me. If I am in need of money, she also gives me money (HBC client of Kara Counselling, Kabwe 1–6).

Home-based caregivers often intervened at crucial moments in the care-seeking trajectories of their clients. Many stories highlighted their involvement with the range of actors involved in illness diagnosis and treatment decisions. For example, in one case, a home-based caregiver described making personal sacrifices to assist their client to reach the clinic. Although likely to have been embellished in retelling, this narrative nonetheless reflects the caregiver’s sense of authority in acting on behalf on their client:

I was compelled to make a personal decision owing to the fact that the husband [of my client] never cared for her and their children were very young therefore, they could not take care of themselves. […] It became my responsibility to support that family. I persuaded her to accompany me so that we could visit a doctor to seek medical help. One day, I woke up as early as 5:00 am, I got my bicycle and went to her place, picked her up and cycled towards the bus stop so that we board a bus heading to the hospital to meet the doctor. […] The doctor followed my request and put this client on ART. I am happy to inform you that this client is still alive, strong and […] she belongs to a support group of ART workers (Home-based caregiver with Dackana, Kabwe 1–1).

A number of home-based caregivers framed their interventions as personal decisions in which they felt morally compelled to ‘encourage’ , ‘persuade’ , and ‘convince’ clients to undertake steps that they were unlikely, or unwilling, to take on their own initiative. In a few stories, this agency extended to eliciting a ‘confession’ from a client, as described by words of one of the home-based caregivers:

He [the client] failed to disclose his HIV results to me, every time I enquired about his medical tests done at the Hospital he was hesitant to talk about it. […] After a long period of time he decided to reveal to me that he was found HIV positive and said *‘it is better I commit suicide’* […]. I encouraged him and told him: ‘*Every human being has a virus… it is only that in some people it is positive while in others it is negative, don’t you worry you can live longer and healthy such that you can even do any type of work that you want*.’ This is how the young man accepted [to start ART] (Home-based caregiver with Kara Counselling, Kabwe 1–1).

These interventions were also facilitated through the home-based caregiver’s privileged access into the clients’ home. Intimate access to clients’ lives and routines allowed for a form of surveillance. Daily or weekly visits of home-based caregivers to their clients’ households were often organised around the necessity to check if the client was complying with prescriptions and scheduled clinic visits:

If, for example, he [client] takes ART at 6:00 am, my job would be to visit him as early 5:30 am pretending that I have just gone there to say ‘good morning’ or to hang around with the client, yet I know that I want to make sure he takes his ARVs (Caregiver with Kara Counselling, Kabwe 3–17).

Caregivers are instructed to observe the client drink the medicine. After drinking the medicine, caregivers have to talk to the client to make sure the mouth is clear and the medicine has been swallowed. Some clients pretend to have swallowed the tablets yet haven’t; they keep tablets under their tongue and wait for the caregiver to leave so that they can spit them out. It is for this reason that caregivers use a technique of interrogating the client immediately after taking their medicine to check whether they have swallowed [the drugs] (Home-based caregiver with Dackana, Kabwe 1–2).

Surveillance of clients on treatment sometimes went beyond monitoring clients’ health status and extended to the realm of patients’ behaviour and family relations. Some caregivers saw it as their duty to provide advice on a variety of issues related to their clients’ lifestyles including relationships, sexual relations, drinking alcohol, smoking, education, and religious values. A few expressed strong opinions about neglectful family members perceived to do too little for the PLHIV:

There are certain families who don’t care. […] There are some situations where the environment is clean but the client is not bathed. So I have to bathe the client or do other chores since you may find the relatives are unconcerned (Home-based caregiver with Kara Counselling, Kabwe 1–3).

Caregivers’ new roles in surveillance and monitoring were viewed by some clients with ambivalence, and seen as a poor substitute for the assistance previously received through HBC. When asked what support they received from caregivers, many HBC clients tellingly understood the question to refer to material subsidies, and replied that there was limited or no support. Their responses hinted at the diversified landscape of ‘support’ for PLHIV in Zambia:

In most cases, she [caregiver] is just encouraging us to take the drugs. Sometimes she promises that she will give us some soya flour, of course she does, but the amounts are not sufficient… we feel we need more because when you are on ART, you need soya […] but they supply us with very little which finishes, maybe after two meals it is finished (HBC client with Kara Counselling, Kabwe 1–5).

I can’t say that I received a lot of care from the caregivers at UCZ [United Church of Zambia]. The people who helped me […] they used to come to wash, cook porridge, make me honey, you understand, they used to do everything […] but them from UCZ, they used to give me cooking oil, *kapenta* [fish] but now they have stopped, there is no food (HBC client with Family Health Trust, Lusaka 1–12).

Clients continued to impose a number of demands on caregivers including financial help as well as material support and food. However, meeting these demands was not always possible. Most caregivers expressed their unease with going to homes empty-handed and some faced open resentment from the HBC clients:

Nowadays our clients are even angry with us, they shout at us. They accuse us that we appropriate food and other material support meant for them. So we tell them to cool down and we tell them that we are not lying when we say that there is no material support that is provided any longer (Home-based caregiver with Family Health Trust, Lusaka 1–14).

While some home-based caregivers resorted to their own means to meet clients’ expectations, others were made to feel guilty for not being able to provide material hand-outs. One caregiver described being verbally abused:

At times they [clients] accuse us and say: *Maybe you are being given food but you just don’t want to give it to us!* (Home-based caregiver with Family Health Trust, Lusaka 1–15).

Many PLHIV continued to see their caregiver as being responsible to provide support as part of the ‘package’ of HBC. Caregivers, on the other hand, remained acutely aware of the basic needs of clients who did not have regular access to food and lack familial support. For these clients, caregivers continued to demonstrate the vital roles of social support, empathy and community solidarity.

## Discussion

HBC programmes were promoted as one of a number of task-shifting initiatives to deal with the shortage of health staff for delivery of HIV care in low-income settings [21]. Threats to these programmes include the lack of standardised interventions and material support, food insecurity, and a decrease in the time available to deal with clients individually [22-25]. Nonetheless, home-based caregivers are one of the groups of lay health workers increasingly drawn into the machinery of ART programmes as these continue to be expanded and further decentralised [26,27].

Linkages between the formal and informal sector, if formalised and supported, can provide a strong basis for the continuum of HIV care [28,29]. In the context of wider ART availability, our study indicates that Zambian home-based caregivers have acquired enhanced roles as intermediaries between formal health staff and PLHIV. They have moved away from ‘traditional’ , hands-on forms of care and support to more medicalised activities that underpin the goals of timely access and sustained adherence to ART. Home-based caregivers appreciate the additional training and knowledge gained through these new roles; our findings confirm that acquisition of professional skills is an important motivational factor for lay caregivers [30-32]. They have become extensions of the health system, are able to alleviate some of the burden of patient tracing, counselling, and follow-up to assist health facility-based health care staff, and play an important role in assisting PLHIV and their families in navigating the HIV care trajectory.

Recent studies assessing the feasibility of task-shifting in HIV care have accordingly focused largely on the technical aspects of transferring activities to lower level health cadres of health care workers, including lay caregivers. They suggest that lower level health care workers, with sufficient training and adequate supervision, can play a key role in providing a range of health services. These include vital signs and anthropometry measurements, counselling, testing, prescribing ART, and adherence support ‘safely’ performed by nurses, lay counsellors, community volunteers and expert patients [9,11-13].

However, our study indicates that home-based caregivers’ ability to adapt to the changing nature of HIV care is not merely a question of knowledge and skills, but of the professional and social relationships they form and sustain, and the recognition gained through these ties. In this setting, despite the upgrading of their tasks, caregivers protest the lack of formal visibility and compensation. At the same time, they seem trapped by notions of volunteerism and altruism that prevail in public discourse and the literature on motivation of lay health care workers [18,22,30,33].

Given the harsh backdrop against which lay caregivers carry out their work, it has been argued that “*government rhetoric* […] *which consists of romanticized and a-historicized constructs that are presumed to be shared by those targeted by the message*” [34] can and should no longer be misused as a moral incentive to rally community members to work as volunteers. Our study supports other research suggesting that the reliance on volunteerism and ‘community spirit’ in under-resourced settings is unsustainable and exploitative [30,34,35].

Lay caregivers’ motivation has been largely explored in relation to gender, grade, training, time in post, workload, working environments [36-39] as well as social recognition, sense of social responsibility and self- efficacy [40,41]. Our study contributes to the growing body of work reflecting on the relational basis of motivation, highlighting the ambiguous and sometimes conflicting positions that CHWs occupy as they are progressively integrated into formal health systems [18,19]; Acquiring specialist knowledge and creating new partnerships with formal health staff within health facilities were central to home-based caregivers’ motivation, however, this did not preclude an expressed desire for external rewards [31] such as incentives, formal payments, and additional material benefits.

Zambian home-based caregivers’ relationships within communities are also changing with the medicalisation of HIV care, as indicated in another recent study from Mozambique [19]. Accordingly, their motivation may well stem from a sense of being able to make a real difference to their clients’ health seeking trajectories. At the same time, a subtle shift in the power dynamic between home-based caregivers and their clients is emerging as caregivers take on roles of monitoring clients’ compliance not only with treatment regimens, but also with ‘healthy lifestyle’ behaviours. HBC clients, on their part, view caregivers’ new roles with some ambivalence, lamenting the loss of nutritional and material subsidies associated with HBC in earlier years. In this context, material support remains as critical to survival as adherence support. Home-based caregivers, because of their organisational affiliations, are seen as potential channels to access food and other resources, giving rise to expectations and tensions in client-caregiver relationships. The transformation of care relations that we begin to explore in this paper requires a more in-depth grounding in the changing landscape of HIV donor funding, and policies implemented for community involvement in HIV care in Zambia. Researchers studying the unintended effects of Global Health Initiatives (specifically HIV funding) on the health system, HRH [6,42], and sustainability of CBOs in HIV care [43] have largely relied on managerial and policy-maker perspectives. In the light of continued discussion on the role of CHWs in supporting HIV care [5], it appears critical to develop multi-layered accounts that bridge macro-level perspectives on policy decisions regarding how best to deliver HIV services with micro-level narratives of HIV caregiving at the ground level.

### Study limitations

The study is cross-sectional and as such, the data reported on here provides a lens on the debates around formalisation of CHWs that have become topical since the mid-2000s. The data was collected at a time when minimum standards for HBC in Zambia had been drafted but not widely circulated [44], and the discussion of formalising a new cadre of Community Health Assistants was on-going [6]. While ethnographic and longitudinal data might have been able to document ‘true’ changes in the role of HBC, the study was limited by a time frame, and a paradigm of qualitative methodology within health services research. Our principal objective was to elicit a timely snapshot of HBC participants’ personal experiences and perceptions of the evolving scope and impact of home-based care in the context of the changing funding landscape for HIV and on-going policy discussions about the role of CHW in Zambia.

Although we carefully selected eligible study sites across urban, peri-urban and rural areas, and carried out a number of visits prior to the start of data collection, data were collected in urbanised districts in Zambia and may not be representative of more isolated areas, in which the access to ART is limited further by the inaccessibility and scarcity of health care facilities. Our study did not claim to be representative of the situation nation-wide; instead we deliberately focused on an in-depth understanding of HBC and care relations through careful selection of sites, and longer involvement with research informants in each study site.

## Conclusions

As illustrated in the study of Zambian home-based caregivers, the shift in roles for lay health workers to support formal HIV care in the era of ART is not always accompanied by the necessary specialised training, supervisory support or opportunities for professional mobility that might be important factors in motivating and retaining these workers. Motivation to provide good care is not only dependent on the acquisition of professional skills and knowledge, but on recognition, positive working environments, and positive relationships with the recipients of care. In assessing the potential contribution of lay caregivers to the HIV care continuum, we need to carefully consider not only the efficiency and quality of functional tasks shifted, but also the relational basis of motivation that presents both opportunities and challenges in the provision of care [45].

The remit of home-based caregivers has had to evolve in order to meet the demands of the health system in retaining patients on ART in care and empowering them to manage their lifelong chronic condition. The extent to which caregivers can support this transition successfully will, however, depend on attainment of professional recognition while retaining trust and solidarity in communities they serve. This is not self-evident: professionalisation may entail a move towards rational and task-oriented care that may undermine an ethos of ‘community care’ that was at the core of the original HBC programmes launched in Zambia more than two decades ago [6,46]. At the same time, the move towards patient empowerment, for example through ‘expert patient’ initiatives, may reinstate an ethos of care that is grounded in self-help and peer support [47] and hence renders the need for home-based support less evident.

As ‘care’ in the form of expanded ART coverage becomes a measure of both donor and public sector accountability, ethical concerns remain as to the enhanced utilisation of lay health care workers in these settings. Traditional notions of volunteerism based on altruistic motivation are being called upon to fill in implementation gaps and to support the existing workforce. In the context of the growing need for sustainable ways to provide care in a transient environment, the legacy of a system overly reliant on ‘community ties’ and familial networks needs an overhaul. While tangible rewards may ultimately be required to boost and sustain caregivers’ motivation, morale, and professional status, the quality of care provided is also highly dependent on how social relations are construed and maintained in the encounter between caregivers and their clients.

## Abbreviations

AIDS: Acquired immune deficiency syndrome
ART: Antiretroviral therapy
ARVs: Antiretroviral drugs
CBO: Community based organisation
CHWs: Community health care workers
HBC: Home-based care
HRH: Human resources for health
KII: Key informant interviews
NGO: Non-governmental organisation
PLHIV: People living with HIV
